# SARS-CoV-2: Two Years in the Pandemic: What Have We Observed from Genome Sequencing Results in Lithuania?

**DOI:** 10.3390/microorganisms10061229

**Published:** 2022-06-16

**Authors:** Lukas Zemaitis, Gediminas Alzbutas, Emilija Gecyte, Dovydas Gecys, Vaiva Lesauskaite

**Affiliations:** 1 Laboratory of Molecular Cardiology, Institute of Cardiology, Lithuanian University of Health Sciences, LT-50162 Kaunas, Lithuania; emilija.gecyte@lsmuni.lt (E.G.); dovydas.gecys@lsmuni.lt (D.G.); vaiva.lesauskaite@lsmuni.lt (V.L.); 2 Laboratory of Translational Bioinformatics, Institute for Digestive Research, Lithuanian University of Health Sciences, LT-50162 Kaunas, Lithuania; gediminas.alzbutas@lsmuni.lt

**Keywords:** SARS-CoV-2, Lithuania, epidemiology, BA.1, BA.2, delta, Omicron

## Abstract

SARS-CoV-2 has spread vastly throughout the word. In this study, we focus on the patterns of spread in Lithuania. By analysing the genetically sequenced data of different lineages and their first appearances, we were able to compare the dynamics of spreading of the lineages and recognize the main possible cause. The impact of emigration patterns and international travel on the variety of lineages was also assessed. Results showed different patterns of spread, and while a vast variety of different lineages were brought in by international travel, many of the viral outbreaks were caused by local lineages. It can be concluded that international travel had the most impact on the spread of SARS-CoV-2.

## 1. Introduction

### 1.1. SARS-CoV-2 Sequencing History in Lithuania

The first case of SARS-CoV-2 in Lithuania was detected in February 2020. At that time, the sequencing of SARS-CoV-2 in Lithuania had not yet been performed, therefore the first samples (one collected in February and two in March 2020) were sequenced at Charite Universitaetsmedizin, Berlin, Institute of Virology (Germany) and published on the world’s largest repository of SARS-CoV-2 genomes—GISAID (Global Initiative for Sharing All Influenza Data) (GISAID ID: EPI _ISL_416741; EPI _ISL_450496; EPI _ISL_450497). The first instance of SARS-CoV-2 sequencing in Lithuania was performed by researchers at the Lithuanian University of Health Sciences (GISAID ID: EPI _ISP_536398). During the following year, additional sequencing was performed by various initiatives of researchers, and in 2020, a total of 641 analyzed sequences were added to the GISAID database from various Lithuanian laboratories.

### 1.2. Sequencing as a National Surveillance Program

Sequencing as part of a national programme was implemented in February 2021 [1]. As a result, more than 30,000 SARS-CoV-2 cases were analysed, and their gene sequences were uploaded to the GISAID database in 2021 [2]. The main body of the national sequencing programme is the National Public Health Surveillance Laboratory (NPHSL), which is under the Ministry of Health (MOH) of Lithuania and was established to conduct laboratory testing of air, water, and food, and the quality other product, as well as providing the laboratory testing required for healthcare of the general public at a national level. In response to the COVID-19 pandemic, NPHSL was requested to organise and coordinate testing and detection for SARS-CoV-2 variants using next generation sequencing (NGS) technology. Currently, the NPHSL coordinates SARS-CoV-2 WGS testing for four Lithuanian institutions that are capable to conduct sequencing analysis: two third level hospitals, Vilnius University Hospital—Santaros klinikos, and the Hospital of Lithuanian University of Health Sciences—Kauno klinikos, and two universities, the Vilnius University and Lithuanian University of Health Sciences [1].

The NPHSL is responsible for providing methodological guidelines for sample collection, preparation and testing, logistics, data analysis, aggregation, reporting, and publication. In collaboration with the Ministry of Health, sequencing data reports produced by the NPHSL enable rapid response to epidemiological COVID-19 changes in the country and provides important information on SARS-CoV-2 variant (VoC) occurrence and transmission routes [1].

Currently, SARS-CoV-2 WGS is performed weekly, with most samples selected at random, paired with a small percentage of samples with targeted selection based on epidemiology, at the four forementioned institutions with NGS capabilities. Depending on the number of positive COVID-19 cases in different regions of Lithuania, prepared SARS-CoV-2 RNA-positive samples are collected from laboratories and distributed to sequencing centres by the NPHSL logistics team. SARS-CoV-2 WGS coordinated by NPHSL accountant for the SARS-CoV-2 sequencing of human samples for surveillance purpose. No other public or private sectors are included in this program.

At NPHSL, the collected sequencing results are combined with patient metadata and screened for “Variants of Concern,” “Variants of Interest,” and other variants that might contain concerning mutations. The data are shared with the National Public Health Centre, which provides contact tracing and control of emerging SARS-CoV-2 variants. NPHSL is responsible for reporting WGS data to the Tessy and GISAID databases and uploading the results to WHO/ECDC and other international institutions. Based on the sequencing results, NPHSL provides recommendations for testing and tracking strategies of potential carrier contacts to the Lithuanian Ministry of Health. As the total sequencing capacity in the country is insufficient for new variant detection before it reaches 2.5 percent outspread, in addition to WGS coordination, NPHSL biologists prepared approximately 300–600 SARS-CoV-2 positive samples per week to be sent-out to the ECDC reference laboratory for WGS testing.

Currently, all four Lithuanian sequencing centres responsible for SARS-CoV-2 NGS library preparation, quality control, sequencing, and genome assembly are using the available low-scale equipment. All centres have different WGS equipment, library preparation protocols, and analysis algorithms. The data obtained are entered into the National Data Management system Palantir [3] and then uploaded to the GISAID database. Once the appropriate virus variants are identified, the data appear in the Palantir system, where regular updates and notifications are available to local epidemiologists. Currently, NPHSL is developing a new national WGS sequencing infrastructure for COVID-19 and the post-covid era that will allow it to meet the needs of the country.

### 1.3. Sample Selection Strategy in Lithuania

The primary objective of the national sequencing program is to representatively sequence SARS-CoV-2 cases for genomic surveillance in addition to routine surveillance. The sample list for the study is generated semi-automatically by retrieving information on positive samples from electronic health records. Efforts are being made to include samples from all regions of the country (10 counties). Outbreaks in certain regions result in more samples being added to the sequencing list. The downside of this strategy is that some samples fall within the sampling frame about a week before they are collected, which negatively impacts the timeliness of sequencing. The main goal of sequencing is to detect a new variant with a 95% confidence interval for the proportion of a given circulating variant when its relative proportion reaches 2.5%. In accordance with ECDC recommendations, the weakly sample size is estimated to be approximately 800 randomly selected samples at a relative 50% precision [4].

In addition to the general sample list, certain samples are included as targeted sequencing. The goal is for these samples to comprise no more than 10% of all samples selected for sequencing while conserving sequencing resources. In the study group, a proportion of samples are collected non-randomly in the context of epidemiological investigations, suspected viruses with altered epidemiological characteristics, etc. Only some of the most informative samples from a single focus are included (not the entire family, group, etc.). For targeted sequencing, the following high priority criteria are used: individuals at higher zoonotic risk (mink farmers, etc.) and immunosuppressed individuals with disease onset for more than 2 months. The medium priority criteria are as follows: reinfections; travel-related cases, especially from regions with a high positive case count; outbreaks and other epidemiological investigation cases, etc.

After receiving the lists of selected samples, PCR laboratories select samples with PCR value below 30 to assure the quality of the analysis, from randomly selected samples and <32 of targeted sequencing. NPHSL couriers collect the RNA samples on dry ice and distribute them to sequencing laboratories. The average number of samples sequenced per week is about 800, depending on the number of positive cases. The average turnaround time is two weeks from positive PCR to sequencing results, variating between one to four weeks depending on the laboratory.

In order to understand why SARS-CoV-2 caused a global pandemic so fast, and to recognize how such viral advancements could be prevented in the future, here we discuss how different SARS-CoV-2 variants spread from the start of the pandemic to the start of February 2022 in Lithuania. Furthermore, we investigate what the main causes of the vast viral transmission were and how it was affected by various international movements.

## 2. Materials and Methods

### 2.1. Collection of Sequences and Initial Processing

31,334 SARS-CoV-2 sequences used for the analyses were downloaded from GISAID, as were available as at 31 December 2021. Limited data on the spread of the Omicron variant are included in the main results because this lineage spread mainly in 2022, a period that is beyond our study interval, but some findings are discussed, and data are provided as a supplement.

Fasta files and metadata were extracted using ncov-ingest (https://github.com/nextstrain/ncov-ingest (accessed on 19 April 2021)). Lineages for all downloaded sequences were assigned using pangolin 3.1.17 (pangoLEARN 6 December 2021, pango-designation v1.2.105) (https://github.com/cov-lineages/pangolin (accessed on 19 April 2022)). General sequence quality evaluation, extraction, and alignment of S protein sequences and variation calling was carried out using Nextclade 1.3.0 [5]. All sequences having more than 1000 undetermined bases were discarded.

### 2.2. Analysis of Individual Lineages

The analyses were dedicated to finding out what could be the earliest source of the different COVID-19 lineages. The following pairwise analysis steps were carried out:

1. The top 30 earliest-sequenced sequences of a particular lineage were taken as templates for a subsequent similarity search. Only Lithuanian sequences were used as templates. A database to search for the closest sequences was created, sub-setting GISAID to the sequences that were assigned by pangolin to a particular lineage.
2. The database was searched for the closest matches against the templates minimap2 v2.24 [6].
3. A search for cut-off from 0.9500 to 0.9999 with a step of 0.0001 was carried out to choose the identity level at which the number closest to the template’s sequences would be less than 12,000.
4. One thousand random sequences were sampled without replacement from the matched sequences with identity ≥ 0.996. Sampling did not include sequences selected in the 3rd step. The additional set of sequences was necessary to compose a sequence set suitable for maximum likelihood dating.
5. Alignment of sequences from the 3rd and 4th steps against the reference COVID-19 sequence was carried out using the augur v.13.1.0 “align” command [7].
6. Based on the alignment, a maximum likelihood tree was calculated by VeryFastTree v.3.1.0 [8] using a generalized time-reversible nucleotide substitution model. Additionally, command line parameters were used to enable us to rescale the branch lengths and compute a Gamma20-based likelihood. The following command line parameters were used (except indicating input/output files): “--nosupport --gamma --nt --gtr --double-precision”.
7. Polytomies of the tree were solved using gotree v0.4.2 [9].
8. Tree refinements interfering with the branch lengths were optimized using RAxML-NG v.1.1.0 [10], as recommended by C. Young et al. [11]. The command line parameters used (except input/output) were as follows: “--blopt nr_safe--redo --precision 12 --model GTR + FO + I + R4 --evaluate”.
9. The tree was further refined and re-rooted, calculating time tree using TreeTime [12] via the augur v.13.1.0 “refine” command 7. Nodes were assigned to their marginally most likely dates, and confidence intervals for node dates were calculated. A command line flag was set to estimate a constant coalescent rate. Additionally, a flag was set to remove tips that deviate more than four interquartile ranges from the root-to-tip vs. time regression. The command line parameters used (except indicating input/output files) were as follows: “--timetree --coalescent opt --date-confidence --root best --date-inference marginal --clock-filter-iqd 4”.
10. The time tree from the 9th step was used to infer ancestral traits using TreeTime via the augur v.13.1.0 “traits” command [7]. The program was run to infer a country state of a node with sampling bias correction flag set to 3. The command line parameters used (except indicating input/output files) were as follows: “--columns country --confidence --sampling-bias-correction 3”.

The tree from the 8th step was modified using the gotree v0.4.2 [9] utilities to contain only tips that were not filtered out while constructing the time tree (9th step), and the root was placed at the same node as it was placed while constructing the time tree. The trees from the 11th step were subjected to detection of transmission clusters by Phydelity v2.0 [13]. Before Phydelity was run, the tree was split into groups of at most 3000 sequences containing monophyletic sub trees, which were used as a direct input for Phydelity. The command line parameters used (except indicating input/output files) were as follows: “--k 2 --collapse_zero_branch_length”.

### 2.3. Additional Calculations and Processing of the Phylogenetic Data

The MRCA (most recent common ancestor) node for the transmission clusters was found using the MRCA function form ggtree v2.4.1 R package [14]. The transmission frequencies from country to country were extracted from the output files produced by the 10th step of the analysis workflow described above. It should be noted that we utilised not the symmetrized transmission frequencies that were used by TreeTime to infer the migration model, but the observed asymmetric transmission frequencies from the tree.

Analysing month-wise data from GISAID, cubic spline interpolation was carried out using the npreg v1.0-8 R package [15]. All distinct data points were used as knots, and a smoothing parameter search was set to be carried out using a method based on Akaike’s Information Criterion. An interval of the earliest and latest months that contained at least one sequence matching a particular lineage was used for the interpolation. Aggregating monthly lineage abundance data from GISAID, the unknown data points at months where a particular lineage was not detected at a particular month but was observed at any earlier and any later months were interpolated using the na.interpolation function from the imputeTS v3.2 R package [16]. Linear interpolation was used. The R0 for lineages was calculated using the R0 v1.2-6 R package [17]. The reproduction number was calculated by a maximum likelihood method. It was presumed that generation time is, on average, ~5 days, and it could be represented by gamma distribution with SD equal to ~2.5 days. 

Making analysis relating transmissions to Lithuania and migration, the migration data were taken from the data logs of the Lithuanian Statistics department [18]. The total emigration counts from Lithuania to foreign countries from 2010 to 2020 were used for analysis. Countries with the lowest immigration counts from Lithuania and phylogeographic transmission estimates were filtered out before correlation analysis between phylogeographically inferred transmissions and the migration data. In the case of transmission cluster data, only countries participating in more than one transmission cluster potentially representing transmissions to Lithuania were analysed. In the case of TreeTime data, countries with observed transmission frequency to Lithuania >0.1 and with >1000 total net Lithuanian immigrants were analysed.

## 3. Results

**Figure 1:**
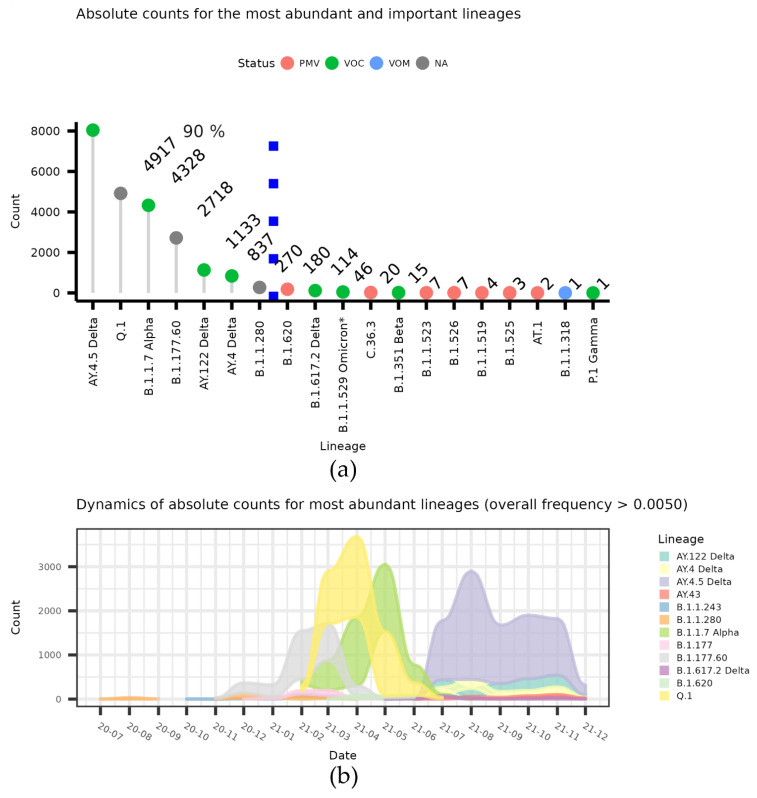
The most prevalent lineages in Lithuania. (**a**) Counts of the most prevalent lineages based on GISAID data by the end of 2021—data on the most frequent lineages that make up 90% of all cases are given (separated by blue box line). “VOC” denotes variants of concern, “VUM” denotes variants of interest/under monitoring, and “PMV” denotes lineages that fell into any of the “special” categories in the past. (**b**) The dynamics of absolute count for the lineages which makes up >0.50% of all cases.

The dynamics of the lineages that make up at least 0.5 counts for the top strains that make up 90% of all Lithuanian sequences in GISAID data, together with other potentially important lineages, are indicated in Figure 1a. The month-wise dynamics of the lineages that make up at least of 0.5% of all Lithuanian sequences in the GISAID data are depicted in the alluvium plots in Figure 1b.

**Figure 2:**
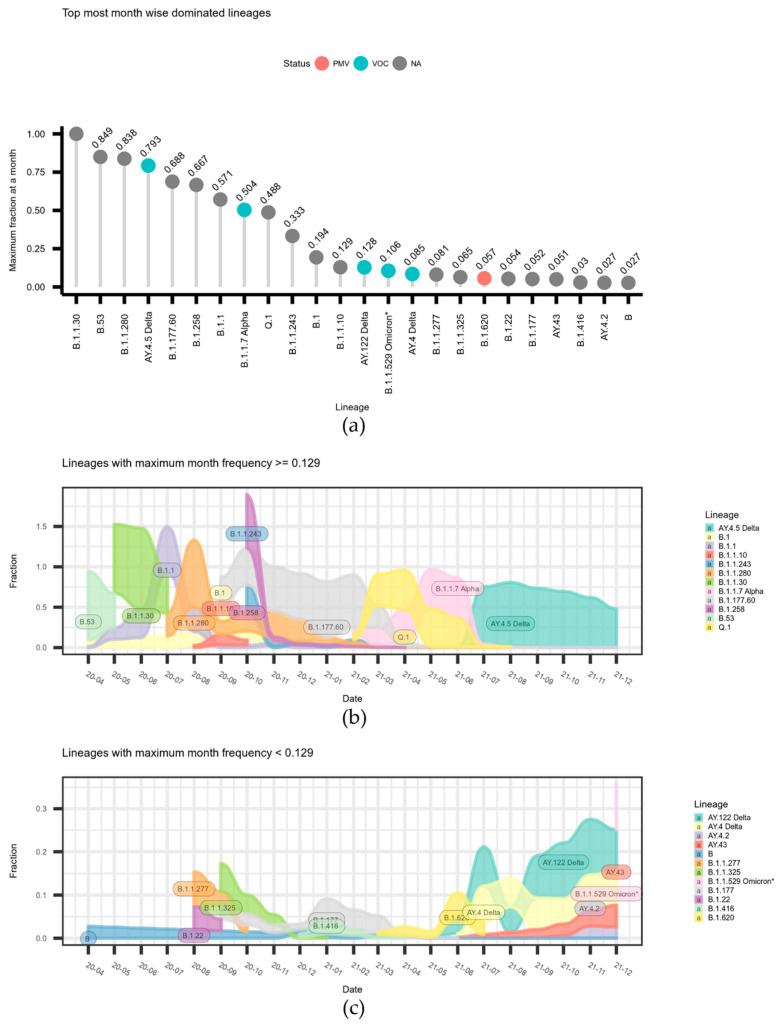
Month-wise dominating lineages (by the end of 2021). (**a**) The lineages ordered in decreasing order based on a maximum monthly frequency in the GISAID data. Top 24 most dominating month-wise lineages are shown. (**b**) The dynamics of the 12 most month-wise frequent lineages. (**c**) The dynamics of the 13–24th most month-wise frequent lineages.

The data on relative month-wise frequency are given in Figure 2. The data on the maximum monthly frequencies per lineages are given in Figure 2a. The data on correspondingly month-wise dynamics are divided in two parts, for the most frequent and less frequent lineages, and are depicted in the Figure 2a,b alluvium plots.

As we see in terms of the absolute numbers of COVID-19 spread (Figure 1), Lithuania was dominated by several Lineages (most notably A.Y.4.5 and Q.1). However, until the autumn of 2020, there was no clear domination of one Lineage (Figure 1a and Figure 2a), and we were then faced with two clear waves: by the end of 2020, B.1.177.60 started a significant spread, and was then overtaken by B.1.1.7 and its localized version Q.1. Both slowly diminished, then a spike in numbers was caused by a Lithuanian Delta variant, A.Y.4.5. As we can see in Figure 2a, the Delta variant A.Y.4.5 dominated over other lineages by the end of June 2021 and was further replaced by other Delta subtypes (Figure 2c).

**Figure 3:**
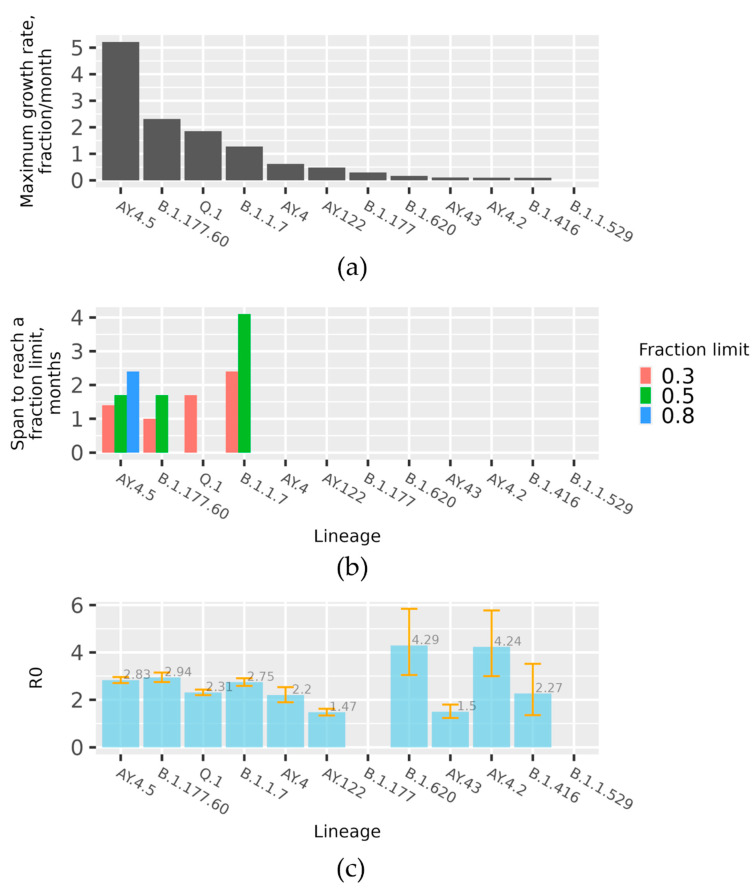
The fastest spreading lineages. The data represent lineages with more than 100 Lithuanian sequences in GISAID. The data presented in (**a**,**b**) are based on spline interpolation of observed month fractions of all GISAID-deposited Lithuanian sequences. (**a**) Plot indicates the maximum first order derivative of the spline fitted to the month fractions of each lineage. (**b**) Plot indicates the time spans required to reach a certain fraction limit of all GISAID-deposited Lithuanian sequences (indicated by fill colour). (**c**) The plot represents calculated R0 values for the lineages based on the absolute number of corresponding lineages aggregated on a monthly basis. For some lineages, it was impossible to calculate R0 values, and they are not given.

The data on the pace of spread for the lineages are represented in three aspects. The data on initial expansion rate of the month-wise fraction increase per lineages are given in Figure 3a. The corresponding modelled R0 values based on absolute counts are given in Figure 3c. Several lineages managed to reach a month-wise fraction of over 30%. The data on the time over which such lineages reached a certain month-wise fraction are given in Figure 3b. As we see in Figure 3a, the fastest observed spread was in the case of AY.4.5 and B.1.177.60 lineages, followed by Q.1. and B.1.17. Only one lineage, A.Y.4.5, managed to reach an 80% month-wise fraction (due to the limited study interval, data with Omicron Spread in early 2022 are not included), B.1.1.7 and B.1.177.60 reached 50% month-wise fraction, and Q.1 reached 30%.

**Figure 4:**
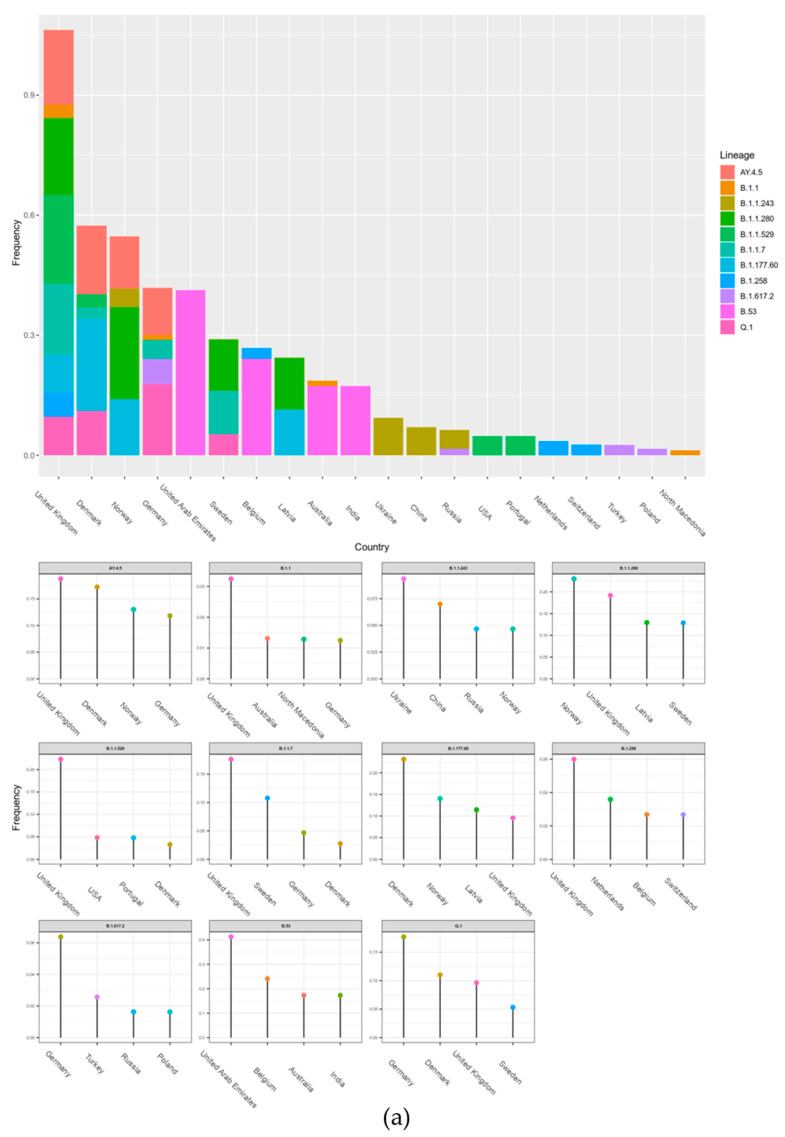

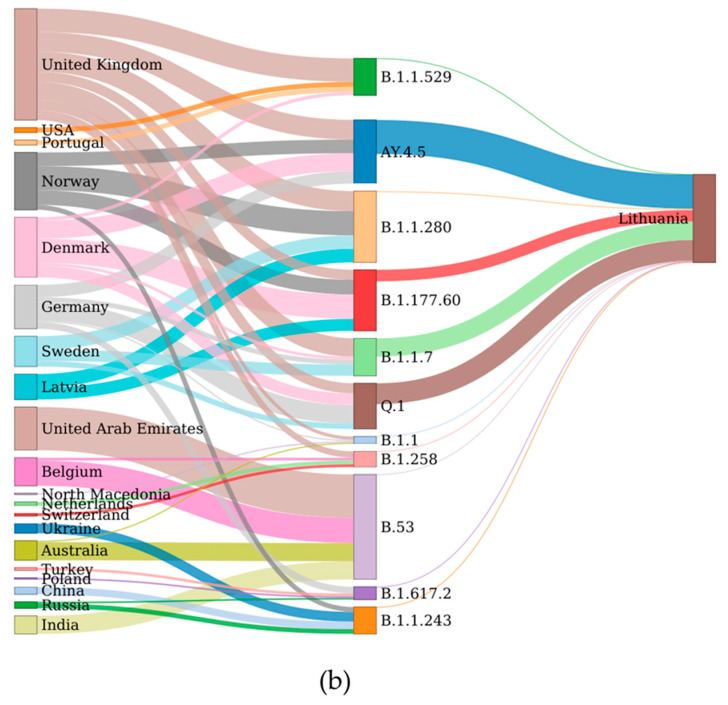
The most likely countries of origin for the most prevalent lineages based on the GTR model. (**a**) The Y axis depict frequency of transmission to Lithuania from the listed countries, as inferred from the time-scaled phylogenetic trees. The upper graph depicts the cumulative frequencies across different lineages. The small graphs below give details of the top four countries with the largest observed transmission frequencies to Lithuania. (**b**) The columns in the middle of the Sankey diagram depict lineages. The widths of the flows to the left side of these columns are proportional to the frequency of transmission to Lithuania, as inferred from the time-scaled phylogenetic trees. The width of the flows to the right side of the columns in the middle are proportional to the fraction of all GISAID-deposited Lithuanian sequences matching a particular lineage.

**Figure 5:**
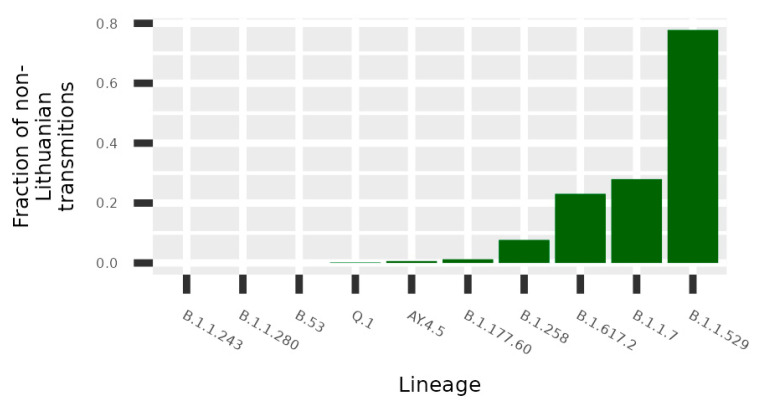
Potential non-Lithuanian origin transmissions. The figure indicates the fraction of sequences included in detected transmission clusters for which the most recent common ancestor is a non-Lithuanian sequence.

**Figure A1:**
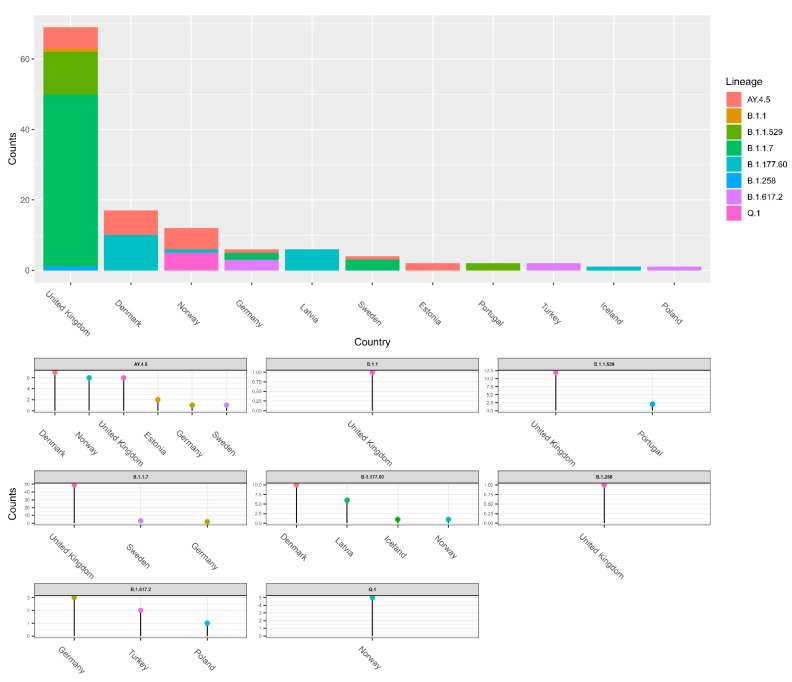
The Most likely countries of origin for the most prevalent lineages based on transmission clusters. The Y axis depict frequency of transmission as to Lithuania from the enlisted countries, as inferred from the phylogenetic trees. The upper graph depicts the cumulative counts of transmission across different lineages. The small graphs below give details of the top four countries with the largest observed transmission counts to Lithuania.

The data of potential transmission to Lithuania were evaluated in two ways. The results on the transmission cluster-based analysis are given in Figure A1, and the results based on the TreeTime are given in Figure 4. In both cases, the upper part of the figures represents aggregated data per country and the smaller graphs give details per different lineages. Additionally, a schematic view of the data shown in Figure 4a is represented in Figure 4b. As we see in Figure 4, the United Kingdom, Denmark, and Norway are notable for the highest transmission intensities to Lithuania; however, the data indicate that some lineages originated from other countries: B.1.1.243 (Ukraine), B.53 (United Arab Emirates), and Q.1 and B.1.617.2 (Germany). Only the analysis based on transmission clusters enabled direct evaluation on rate of non-Lithuanian origin transmissions compared to local origin transmission (Figure 5). The data indicate that the B.1.1.529, B.1.17, B.1.617.2, and B.1.258 lineages stand out as lineages with the highest fraction of potential foreign origin transmissions (listed in decreasing order).

**Figure 6:**
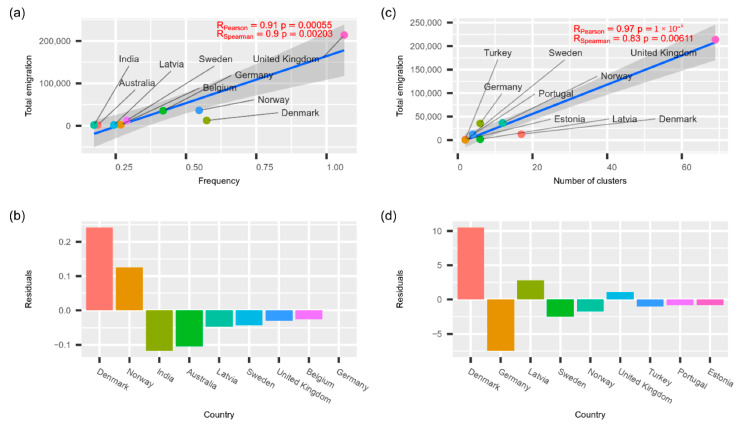
Relationship between total emigration rate (data up to 2020) and potential country of origin, as deduced from the phylogenetic analysis. Plots (**a**,**b**) depict data based on transmission frequencies inferred from time-scaled phylogenies. Plots (**c**,**d**) depict corresponding data inferred from transmission cluster analysis. The residuals from the linear model in plots (**a**,**d**) are depicted in decreasing order. The transmission measures were summed across all analysed lineages before calculating the correlations.

The results of correlational analysis between known emigration data and transmissions to Lithuania are given in Figure 6. As we see in the figure, phylogeographical estimates of foreign transmission intensities are directly proportional to the total emigration intensity from Lithuania within the last ten years, and corresponding Pearson and Spearman correlation estimates are statistically significant. It is worth noticing that, based on the residuals of the linear models, Denmark and, to a lesser extent, Norway stand out as having higher transmission intensity to Lithuania than expected. The higher-than-expected transmission intensity from Germany is evident despite the way we analysed phylogeographic transmissions. However, higher-than-expected transmissions from Norway are only evident using transmission cluster-based estimates. Correspondingly, India and Australia are notable for lower-than-expected transmission intensities (based on the TreeTime GTR model), along with Germany (based on transmission cluster estimates).

**Figure 7:**
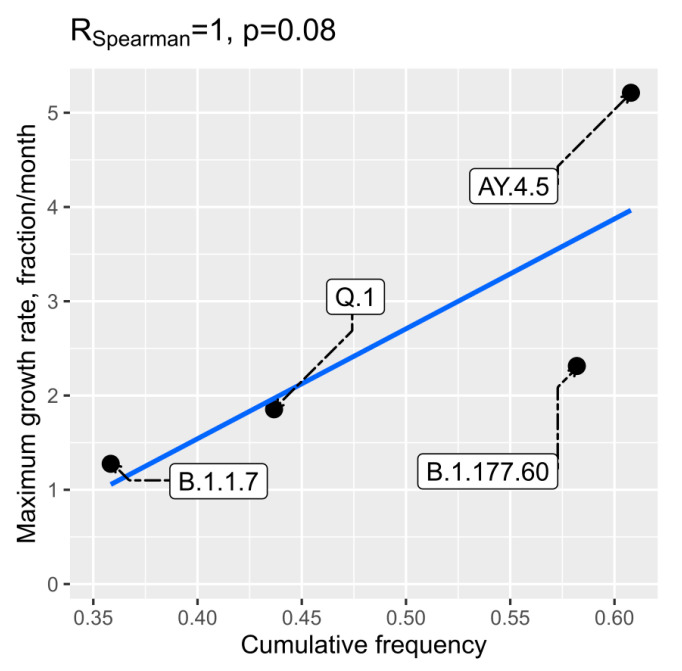
Relationship between frequency of transmission to Lithuania, as inferred from the time-scaled phylogenetic trees, and initial growth rate. On the x-axis, the sum of transmission frequencies from abroad to Lithuania, as inferred from the time-scaled phylogenetic trees, is given, and the y-axis matches the maximum first order derivative of the spline fitted to month fractions of the lineages.

We also checked if higher evaluated R0 values or initial spread dynamics could be related to higher transmission intensities from abroad to Lithuania. Pearson and Spearman correlations between frequency of transmissions to Lithuania and R0 were low and statistically insignificant. However, we detected a weak correlation between initial maximum growth rate and frequency of transmissions to Lithuania as inferred from the time-scaled phylogenetic trees. The data on both of these two aspects were available only for four lineages and are given in Figure 7. The P value for the Spearman correlation was 0.08 (R = 1).

## 4. Discussion

During the pandemic’s first year (2020), several viral lineages were distinguished and were all prevalent at the same time. By the end of the second year of the pandemic (2021), the viral advancement pattern had changes, showing one or more dominant lineages, instead of the high variety noted previously. 

The first SARS-CoV-2 genomes that were sequenced in Germany (n = 3) had a very similar gene sequence to the Wuhan-hu-1 [19] lineage, differing by an average of 4–5 nucleotides [2,20]. It is noteworthy that in one of the first cases in Lithuania that were sequenced, a strain with the S:D614 mutation was detected, which was later confirmed to have higher infectivity and promote a faster spread [21,22].

In April 2020, the first B.53 virus lineages were identified in several samples collected and analysed in Lithuania. This lineage was first detected in February 2020 in the United Arab Emirates and is characterized by S:Y28H, ORF:8S84L, and N:P344S mutations [23]. According to GISAID data (February 2022), a total of 84 cases were registered worldwide, of which 73.0% were in Lithuania, 24.0% in the United Arab Emirates, 1.0% in India, 1.0% in Belgium, and 1.0% in Australia [2,23]. It is likely that this lineage was responsible for the first major outbreak that began to be monitored in the second half of March 2020. Due to the low amounts of sampling, it is difficult to evaluate the prevalence of this viral lineage; however, we have observed a wide spread of B.53 at the beginning of the pandemic. In the following spring and throughout the summer, two other Lithuanian SARS-CoV-2 lineages (B.1.1.30 and B.1.1) started to spread. 

The second widely viral lineage was B.1.1.30, which spread during the period 18 May 2020–3 June 2020. All 13 cases of this lineage were registered in Lithuania, Vilnius district. With a gradual decrease in the number of cases with this lineage, Pango, classified as B.1.1, which is characterized by mutations in S:D614G, N:G204R, N:R203K, NSP2:T332I, and NSP12:P323L, spread at a similar time. It comes as no surprise that both the B.1.1.30 and B.1.1 lineages’ most intensive spread to Lithuania was from the United Kingdom (Figure 4), which is the country that hosts most Lithuanian emigrants. The outbreak of this lineage was replaced by the vast spread of B.1.1.280. The viral lineage, which has been present in Lithuania for almost a year, was observed from 30 July 2020 to 12 July 2021, with the percentage of cases being 92.0% in Lithuania, 3.0% in Norway, and about 1.0% in Germany, the United Kingdom, and Latvia [23]. These figures agree with the data in Figure 4 indicating that the most intensive spread of this lineage to Lithuania was from Norway. This lineage was not related to the changes in the biological characteristics of the virus; therefore, it is likely that the epidemiological factors, such as long-term spread, were the main cause of the wide spread of this strain, rather than the altered viral characteristics.

The increase in variety of lineages could be related to summer holidays or similar travels. The first more-pronounced mutation of a single virus line was observed in late autumn, when the number of COVID-19 cases began to rise rapidly. The prevailing Lithuanian virus lineages resulted from the Spain-originated lineage B.1.177, which at that time was the dominating lineage in Europe. Currently, there is limited evidence of increased transmissibility or severity of the infections associated with the B.1.177 lineage [24]. The fact that B.1.177 was defined by the S:A222V mutation in the Spike protein could also confer a significant advantage to the virus infectivity, favouring its rapid selection and dissemination. B.1.177.60 was the first lineage to over-dominate others, and in September of 2020 this lineage was observed in Lithuania for the first time. There is no evidence of any appreciable effect of A222V on the phenotype of the virus (i.e., infectivity and transmissibility), and thus it is generally assumed that its increase in abundance was random rather than a selection advantage [25]. As we can see in Figure 3, this lineage is notable for very fast initial spread rate—it is next to AY.4.5 (Delta sub-lineage), and within a little more than one month and a half this lineage reached 50% of all month-wise sequenced cases. Throughout the propagation period, eight sublines were identified, of which the most spread-out was the Lithuanian subline B.1.177.60, which had appeared quickly after the emergence of B.1.177, which had its last cases in Lithuania observed on 21st of November 2021. As we can see in Figure 4, this lineage spread to Lithuania is linked with Nordic countries (Denmark, Norway). Since the lineage was discovered, n = 5162 sequences were produced from sampled cases worldwide. The mutations present in this sub-lineages spike protein were S:L18F and ORF3a:V77F, in addition to the conventional B.1.177 mutations ORF1b-P314L, S:A222V, S:D614G, ORF8:S84L, ORF10:A220V, and ORF10:V30L. Although B.1.177 has been introduced to Lithuania many times, from the profile of hereditary mutations we can say that the vast majority of cases caused by the currently circulating B.1.177 lineage originated from several larger outbreaks. It is the first virus lineage of Lithuanian origin that is widely distributed abroad (mainly in Northern Europe), with the total percentage of cases being 57.0% in Lithuania, 14.0% in Latvia, 12.0% in Denmark, 6.0% in Finland, and 3.0% in Estonia [23]. Thus, the trend of local spread remained the same. In 2021, in February, the peak of this lineage was reached, and with the introduction of the first variant of concern (VOC) B.1.1.7, also known as Alpha, it began to advance this over-dominated lineage.

**Figure A2:**
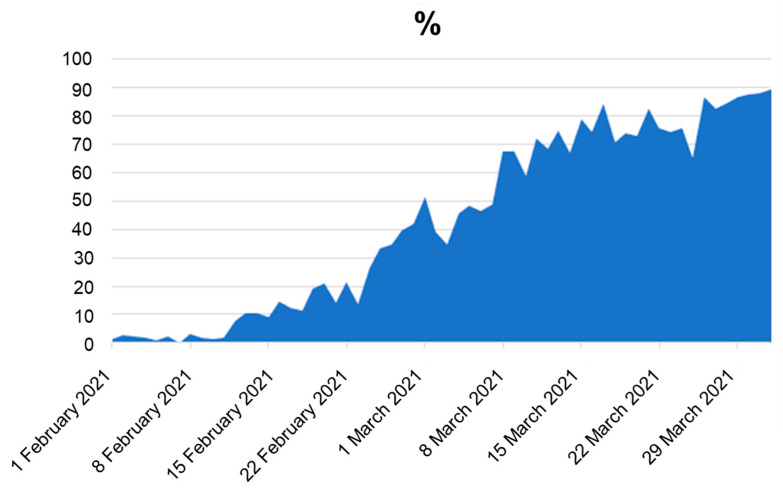
S-dropout due to H69-70del in the Alpha variant.

**Figure A3:**
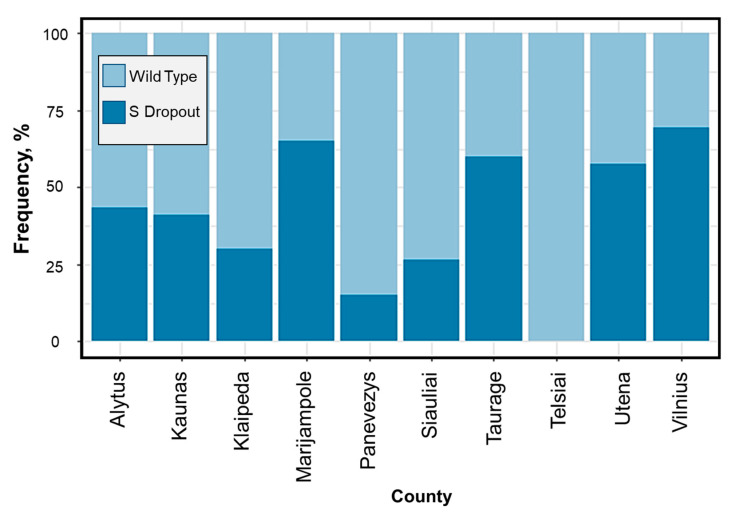
Marijampole, Taurage, Utena, and Vilnius counties S-dropout accounted for the largest spread >50%; in Alytus and Kaunas counties, S-dropout samples accounted for about 40% of the samples, Kaipedos about 30%, Siauliai 25%, and Panevezys about 12%; in Telsiai county, no S-dropout samples were detected (n = 939).

B.1.1.7 (Alpha variant) was first identified in the United Kingdom in late 2020 and was temporally associated with an increase in regional infections, and the spread to Lithuania is also linked with the United Kingdom. This variant contains more than a dozen mutations when compared with other circulating lineages, having several of them within the spike protein. Subsequently, it became the predominant variant in many countries, until the emergence of the Delta variant. In Lithuania, since the first case of B.1.1.7, a very rapid spread has been observed, with the number of sequenced samples of B.1.1.7 doubling in amount every two week in Lithuania, while the Alpha variant was distinguishable based on the failure to amplify the S gene fragment (S-dropout), caused by S:H69-70del [26] (Figure A2). Due to the delay of sequencing results and being aware of the new VOC, the Lithuanian government decided to take a “snapshot” of detecting B.1.1.7 distribution across the country by collecting and analysing all samples at a high priority rate. According to the results of the research, it was observed that, in Vilnius, Utena, and Marijampole counties, this variant accounted for 70% or more of new cases at that time, and in the other part of Lithuania its prevalence was at least 25% (Figure A3). According to the Lithuanian department of statistics, the regions of Lithuania where the B.1.1.7 virus lineage was predominating led in terms of both cases of illness and hospitalization in the country. At that time, infections with COVID-19 in Vilnius County accounted for 54% of all cases in Lithuania, and the number of cases diagnosed doubled since February. Thus, it is clear that this lineage has contributed significantly to the increase in the number of local infections. The situation was the opposite in western Lithuania, and it is likely that such regional differences were due to restrictions on movement between municipalities. Q.1 (Lithuanian sub-lineage) was pushed out of the country not long after its parent B.1.1.7, which spread rapidly due to frequent internal outbreaks. Q.1 is a local version of B.1.1.7 n. This lineage circulated and dominated before other lineages until the appearance of Delta in June 2021, which, one month later, accounted for 95% of all sequenced samples.

The Delta lineage accounted for more than 90% of all virus lineage detections for 6 months. During this period, AY.4, AY.122, AY.111, and AY.36—different delta sub-lineages—were the most observed, of which variants of sub-lineage AY.4 accounted for the largest percentage during the whole period. The Delta AY.4.5 local lineage out-dominated other Delta sub-lineages. In contrast to the case with Q.1 and its parental lineages, AY.4.5 differs from its predecessor lineages by a mutation in the S protein, S:D1259Y. Even though this mutation has not been fully characterized, there are possibilities that it provided infectivity advantages for the virus [27]. This could be supported by a slightly higher R0 value compared to A.Y.4. However, after its peak, the fraction of AY.4.5 cases started slowly decreasing, while other Delta sub-lineages started to increase. Hypothetically, if the Omicron variant would have never arisen, we would have different Delta sub-lineages coexisting at a similar measure.

**Figure A4:**
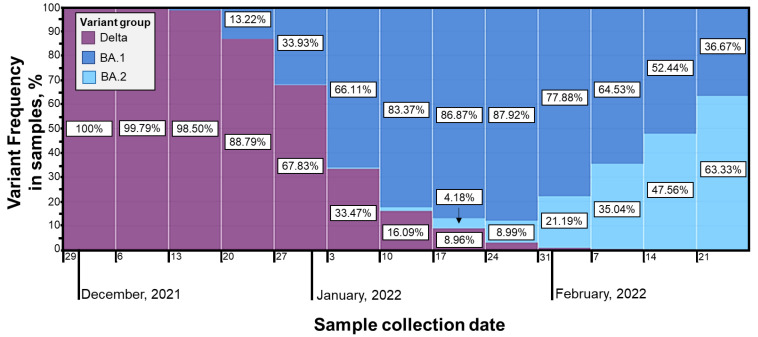
Delta and Omicron subtype changes in Lithuania. GISAID sequences from February 2020 to February 2022.

Omicron was first identified in December 2021 and quickly became the dominant strain. The BA.1 Omicron subtype was the fastest growing in Lithuania, following with a vast increase in BA.2, observed at the end of January and increasing by about 10% each week, leading to rapid over-domination [28] (Figure A4).

Many lineages of SARS-CoV-2 imported to Lithuania from abroad did not spread throughout the country, as shown in Figure 2 and Figure 5; Q.1 and its paternal lineage B.1.1.7 being the exceptions. At the beginning of its arrival/detection, Q.1 rapidly started to spread, and the number of cases grew even faster than the parental Alpha variant (Figure 3). However, it was noted that B.1.1.7 became more prevalent and replaced the previously dominating Q.1 sub-lineage. It is highly likely that the lifting of certain pandemic and travel restrictions in the country is the reason behind the spread. Therefore, we can conclude that travel restrictions have a considerable influence on the spread of various SARS-CoV-2 sub-variants. However, it is worth mentioning that it has a minimal effect on the viral sub-lineages that were imported the most, such as B.1.1.529, B.1.617.2, and B.1.258, due to the fact that they did not contain distinguished traits that could increase their ability to spread. At the time the Delta variant became present in Lithuania, the AY.4.5 sub-lineage started to spread rapidly, influenced by large local outbreaks; however, since July the number of cases has declined compared to other sub-lineages. AY.4.5 is unlikely to have a significant biological advantage, with additional mutations in S:E156G and S:D1259Y in the SARS-CoV-2 genome. Large initial growth at the beginning of the lineages presence was determined as a result of the founder effect and other Delta mutations. 

While analysing the variety of lineages, we noticed that increased traveling during the summer holidays and the easing of quarantine restrictions affected the diversity of the different lineages. We decided to verify this hypothesis by comparing the number of emigrants in the country with countries from which different virus lineages were usually imported by relying on the assumption that the countries that most Lithuanian people have emigrated to would import the highest number of different lineages. This notion was confirmed, and we discovered that the amount of emigration significantly correlated with the amount of different lineages present. However, Denmark was an exception, as emigration to this country from Lithuania is rather small, but the number of observed transmission clusters was rather high. In the present study, we did not investigate this further; however, this instance could be related to the pandemic restrictions and their early easing in Denmark [29].

Lithuania and other countries that recently joined the EU have high emigration rates. We could expect that countries with the most abundant Lithuanian diaspora could be the most prominent sources of COVID-19 transmissions to Lithuania. The data in Figure 4 suggest that this presumption is correct. Both methods used to detect international spread showed that the intensity of spread to Lithuania is directly proportional to the number of Lithuanian immigrants.

It is worth noticing that Denmark-related transmissions to Lithuania are more significant compared to what you could expect based on the size of Lithuanian diaspora. This could be related to the fact that Denmark was the first European country to remove COVID-19-related restrictions [30]. On the other hand, countries such as India and Australia contributed to transmissions to Lithuania less than expected. This could be due to much higher distance and more restricted travel arrangements to these countries compared to other EU member.

We also hypothesized that higher evaluated R0 values or the characteristics of initial spread dynamics could be related to higher transmission intensities from abroad to Lithuania. However, the analysed data allowed us to detect only weak positive potential correlation between maximum infection growth rate and frequency of transmissions to Lithuania (Figure 7). The relationship could be logical, as a more intensive transmission to Lithuania could result in a bigger number of initial spreaders.

## 5. Conclusions

For the most part, the Lithuanian COVID-19 outbreaks were driven by lineages of local origins. A sizeable number of virus lineages has been identified a few times but has disappeared over time. Dominating VOC’s had a significant effect in reducing the diversity of the common COVID-19 viral lineages, but even lineages with more dangerous properties have disappeared without recovery, for example, the B.1.1.523 SARS-CoV-2 variant containing multiple S protein mutations associated with immune escape [31] or B.1.62 [32]. Meanwhile, the spread of viral lineages without altered biological characteristics has been observed more than once, possibly due to uncontrolled outbreaks. Domination of some lineage could be determined by the initial outbreak. It is illustrated by the domination of the AY.4.5 lineage over other Delta sub-lineages, which could be due to the introduction of quarantine, has significantly reduced the variety of lineages. As of now, the Omicron BA.2 subtype is dominating in all regions of Lithuania, after the pushing out of the previously widespread BA.1.1 sub-type (out of this study’s main analysis interval). The analysis of data indicated that the initial spread rate of a new lineage could be related to its transmission intensity from abroad. Our study suggests that international transmissions to Lithuania are predominantly from countries with the largest Lithuanian diaspora population. In the case of novel outbreaks, it would be wise to differentiate travel restrictions, taking into consideration the size of diaspora in other countries, focusing the tightest restrictions on travel from countries with the largest ones. Additionally, it could be suggested that, in the case of the ongoing epidemic, changes in preventive measures in other EU countries should be on constant surveillance considering additional travel restrictions.

## Data Availability

All genome sequences and associated metadata in this dataset are published in GISAID’s EpiCoV database. To view the contributors of each individual sequence with details such as accession number, Virus name, Collection, date, Originating Lab and Submitting Lab and the list of Authors, visit https://doi.org/10.55876/gis8.220614so.
